# Trail using ants follow idiosyncratic routes in complex landscapes

**DOI:** 10.3758/s13420-023-00615-y

**Published:** 2023-11-22

**Authors:** Robert Barrie, Lars Haalck, Benjamin Risse, Thomas Nowotny, Paul Graham, Cornelia Buehlmann

**Affiliations:** 1 School of Life Sciences, University of Sussex, Brighton, BN1 9QG UK; 2 Institute for Geoinformatics and Institute for Computer Science, University of Münster, Heisenbergstraße 2, 48149 Münster, Germany; 3 School of Engineering and Informatics, University of Sussex, Brighton, BN1 9QJ UK

**Keywords:** Wood ants, Navigation, Pheromone trails, Visual navigation, Idiosyncratic routes

## Abstract

A large volume of research on individually navigating ants has shown how path integration and visually guided navigation form a major part of the ant navigation toolkit for many species and are sufficient mechanisms for successful navigation. One of the behavioural markers of the interaction of these mechanisms is that experienced foragers develop idiosyncratic routes that require that individual ants have personal and unique visual memories that they use to guide habitual routes between the nest and feeding sites. The majority of ants, however, inhabit complex cluttered environments and social pheromone trails are often part of the collective recruitment, organisation and navigation of these foragers. We do not know how individual navigation interacts with collective behaviour along shared trails in complex natural environments. We thus asked here if wood ants that forage through densely cluttered woodlands where they travel along shared trails repeatedly follow the same routes or if they choose a spread of paths within the shared trail. We recorded three long homing trajectories of 20 individual wood ants in their natural woodland habitat. We found that wood ants follow idiosyncratic routes when navigating along shared trails through highly complex visual landscapes. This shows that ants rely on individual memories for habitual route guidance even in cluttered environments when chemical trail information is available. We argue that visual cues are likely to be the dominant sensory modality for the idiosyncratic routes. These experiments shed new light on how ants, or insects in general, navigate through complex multimodal environments.

## Introduction

Many animals show idiosyncratic paths when moving through familiar natural environments (monkeys: Di Fiore & Suarez, 2007; pigeons: Biro et al., 2004; ants: Kohler & Wehner, 2005; Mangan & Webb, 2012; Wehner et al., 1996), which suggests that individual animals learn a unique combination of sensory information to guide their habitual routes. In recent years, high-fidelity tracking alongside reconstruction of the sensory information available to animals has allowed us to start understanding the challenges that animals face in real-world navigation and how learning is adapted to natural environments (Freas et al., 2019).

The development and following of idiosyncratic routes requires rapid learning of a large amount of spatial information, which is often but not exclusively visual information (Baddeley et al., 2012; Collett, 2014; Wystrach et al., 2012; Zeil, 2012). This may bring a high computational burden; however, it will subsequently allow individuals to navigate efficiently via relatively simple reactive responses to remembered sensory cues along their routes (Collett et al., 1992). Furthermore, it avoids the need for navigational strategies that rely on integration of information into map-like representations (Hoinville & Wehner, 2018).

Another way to successfully navigate through natural worlds is to use socially transmitted spatial information that is shared between individuals. Collective behaviour allows animals to benefit from spatial information that is socially transmitted, for example, the waggle dance in bees (von Frisch, 1967), following pheromone trails in ants (Aron et al., 1993; Rosengren & Fortelius, 1987), tandem runs in ants (Franklin, 2014; Hölldobler et al., 1974), or group navigation in pigeons (Sankey et al., 2022).

Social insects such as ants allow us to study the interactions between social and individual navigational behaviours. Social cues, such as pheromone trails, and individual cues, such as visual cues, are ideally used simultaneously (Evison et al., 2008), but the interactions and balance of cues change with an ants’ foraging experience. Many experiments have shown that ants follow pheromone trails when they are naïve (Harrison et al., 1989), but that private information is prioritised in experienced ants when social and individual information is experimentally put in conflict (Grueter et al., 2011; Harrison et al., 1989). Odour trails are useful for ants that are inexperienced or unfamiliar with an environment, because such cues can act as a scaffold for individual route learning (Collett et al., 2003). If ants are familiar with an environment, however, they often use individually acquired spatial information (Collett et al., 2013). What we do not know, however, is how social information and individually acquired spatial knowledge interact in natural navigational behaviours in the wild.

Wood ants navigate through cluttered woodlands where they feed on honeydew from aphids on trees that are up to 100 m away from their nest (Domisch et al., 2016; Rosengren et al., 1979). The ant foragers travel along shared odour trails, and previous field experiments have shown that individual wood ants show site-fidelity or *ortstreue* (Rosengren, 1971; Rosengren & Fortelius, 1986; Salo & Rosengren, 2001), which means that they specialise on foraging to a particular place and may have the opportunity to develop idiosyncratic routes, even when collective trunk trails exist. Learning and following idiosyncratic routes would mean that ant foragers need to learn environmental information to guide these routes. We have gained extensive knowledge about wood ants’ navigational skills from experiments performed in the lab, and this strand of research has revealed that these ants are excellent visual navigators (e.g., Buehlmann et al., 2016; Graham et al., 2003; Harris et al., 2005; Lent et al., 2010). We do not know, however, what navigational strategies they are using when navigating through natural woodlands. Here we ask if wood ants learn and follow individually learnt idiosyncratic routes as they move along shared odour trails.

## Methods

### Ants and field site

Experiments were performed with wood ants *Formica rufa* in Abbot’s wood, East Sussex, UK. All data were collected between June and August 2022, from a single nest.

### Experimental procedures

**Fig. 1:**
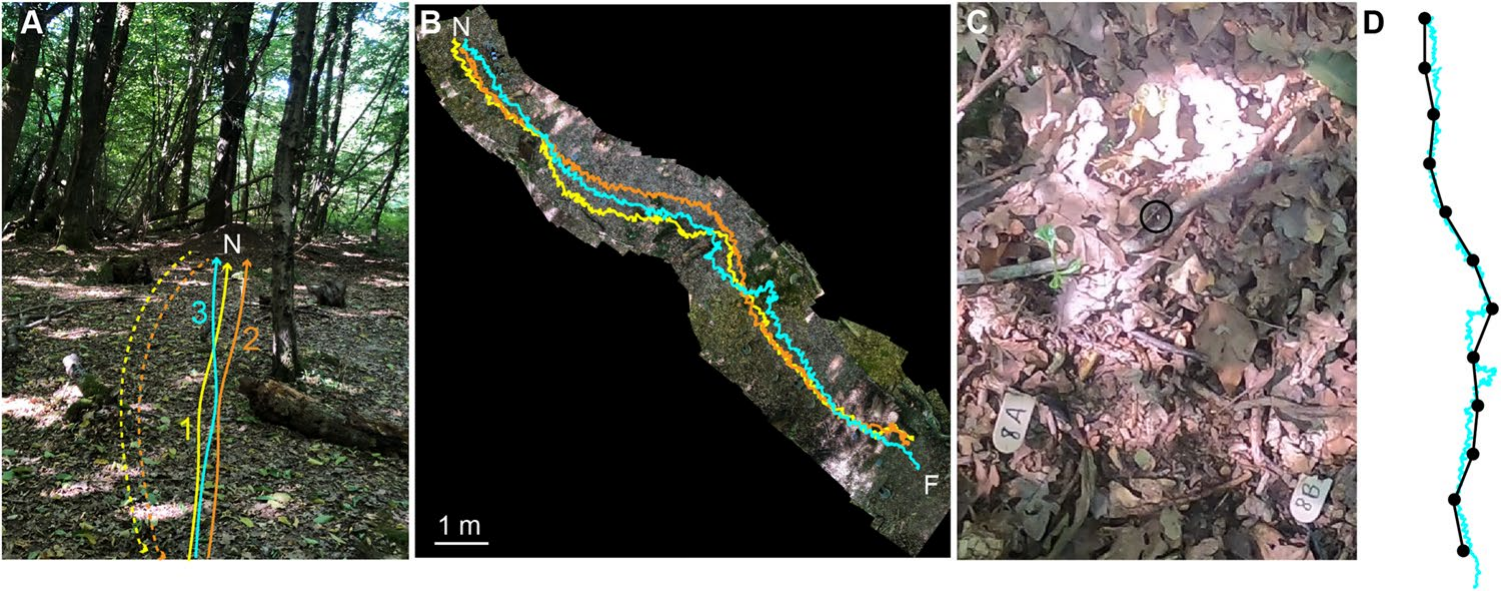
Experimental procedures. (**A**) Three consecutive homing routes of individual ants travelling from the tree to their nest (N) were filmed with a handheld camera. Solid lines: First, second and third homing routes of an individual ant. Dashed lines: When an ant arrived at the nest, we carried her back to the tree to record another homing route. (**B**) Combined animal tracking and environment reconstruction algorithm (CATER; Haalck et al., 2023) showing three homing routes of an individual ant. N, nest. F, foraging tree. (**C**) Area of a video frame showing the tracked ant (marked with the black circle) on the cluttered woodland ground, ground markers are also visible in the picture, with the distance between the two wooden markers placed on the ground being 20 cm. (**D**) For path analysis, we extracted the coordinates of the ants’ paths from the videos using the markers on the woodland ground (x axis: 0.2 m intervals; y axis: 1 m intervals). A single trajectory with high temporal resolution (shown in blue; extracted using CATER) is overlaid with the path plotted using the extracted coordinates (shown in black)

We recorded three consecutive homing paths of individual wood ant foragers that had fed on honeydew from aphids on a tree where this colony was foraging (Fig. 1A). The tree-to-nest distance was 12.5 m. To easily localise and identify individual foragers on the cluttered woodland ground, we marked foragers coming down the tree with paint. To do so, homing ants were carefully taken from the tree trunk approximately 1.5 m above ground, marked with a small amount of white paint, and then put back at the same location we took them from. More specifically, we marked a group of approximately five ants and waited for them to arrive at the base of the tree. The first one arriving at the base of the tree was then filmed individually with a handheld camera (Gopro 8 attached to a Feiyu Tech gimbal) from the moment the ant touched the ground until it had reached the proximity of the nest (first homing route). When the ant arrived at the nest, we captured the ant and released it at the base of the tree and filmed its homing path again (second homing route). When the ant reached the nest again, it was taken back to the base of the tree for a third time and a final homing route was recorded from the same ant (third homing route). Recording multiple homing paths of individual ants is a well-established method to investigate how different navigational strategies are used (e.g., Collett et al., 1992; Kohler & Wehner, 2005; Wehner et al., 1996; Wystrach et al., 2019) because for each route repetition the path integration state of the animal is different but the environment stays the same. Only one ant was recorded at a time, and this procedure was repeated until we had recorded 20 individual ants.

We recorded data about the specifics of the routes in two ways. Firstly, we placed small wooden markers (approximately 1 cm × 2 cm in size, see Fig. 1C) on the ground in regular intervals, every 1 m along the direct tree-to-nest path (y axis for plots and analyses) and every 0.2 m perpendicular to it (x axis). This created an approximately 5-m wide grid from the tree to the nest. These markers allowed us to extract the coordinates of the ants’ paths from the videos at 1-m intervals along the tree-to-nest path. Secondly, for some paths, we also tracked the homing paths using a recently developed combined animal tracking and environment reconstruction toolbox named CATER (Haalck et al., 2023, Figs. 1B and D). CATER is capable of detecting small animals against complex natural environments while embedding their movement paths on an environment reconstruction. It uses an unsupervised tracking framework for accurate detection and a reconstruction algorithm based on image-mosaics (Haalck et al., 2023).

In addition to the ants’ homing paths, we recorded panoramic images at marker locations with a Kodak SP360 4K Explorer Pixpro Action Camera that was placed on the ground to provide the “ant’s eye view”.

### Data processing and analysis

To address the question of whether wood ants follow idiosyncratic routes, we simplified the paths by extracting coordinates for 1-m intervals. Paths were defined by their coordinates every metre along the y axis, from 1 m to 12 m along the tree-to-nest distance. If ants crossed a marker directly or very close by, the x coordinate for the marker location was used. If the ants crossed between two markers (0.2 m apart), the x value for the mid-point of that range was taken. On the rare occasion that ants made a small turn and crossed a 1-m line twice, we took the ants’ first crossing. To test for idiosyncrasy in homing routes, we calculated for every ant how similar its three paths were to each other (intra-ant comparisons). That means that we calculated for each ant the inter-path differences for the three route pairs (first vs. second, first vs. third, and second vs. third). Inter-path differences were calculated as absolute differences in the x coordinate for each metre along the y axis, and the mean inter-path difference was then calculated as the average absolute x-difference along the paths. Additionally, we calculated these differences for all inter-ant trajectory pairs. Intra- and inter-ant comparisons were compared using a Mann Whitney test. All path analyses were done in Matlab.

Panoramic pictures were downsampled using the imresize function in Matlab to show the 360° view with 4° resolution in black and white, thus resembling to some extent the ant’s perspective visual scene (Schwarz et al., 2011; Zollikofer et al., 1995).

## Results

**Fig. 2:**
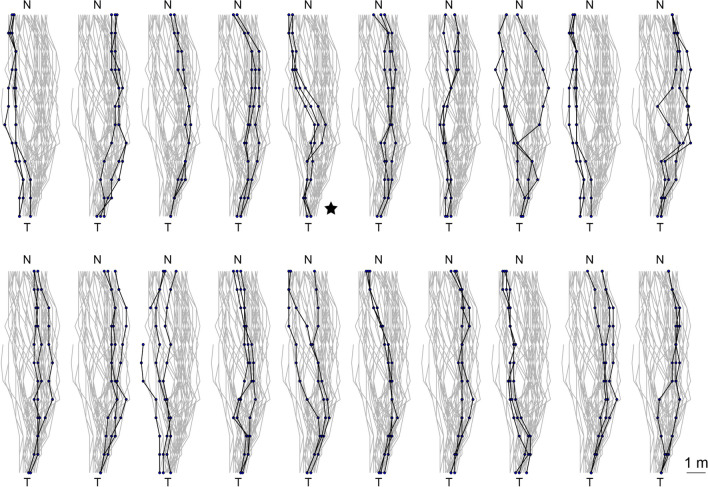
Idiosyncratic routes from homing ants travelling from the tree (T) to their nest (N). Three consecutive homing routes were recorded for all 20 ants. Grey lines show the total 60 homing routes and black lines show the three routes for each individual in turn. Black filled circles show path coordinates every 1 m along the tree-to-nest distance. The ant from Fig. 1B is marked with an asterisk

**Fig. 3:**
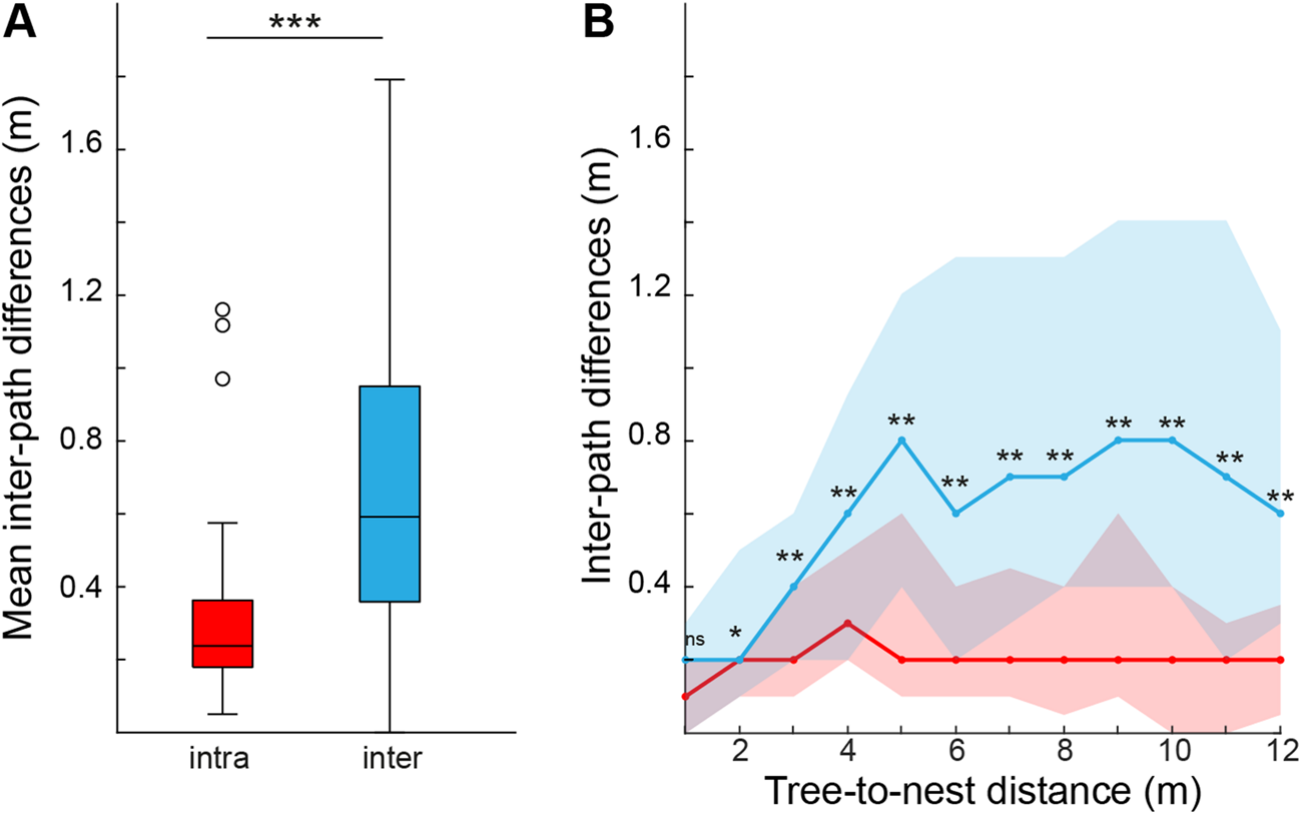
Paths from individual ants are more similar to each other than to routes from the other ants. (**A**) Mean inter-path differences for intra- and inter-ant comparisons. Intra-ant comparisons (n = 56) and inter-ant comparisons (n = 1,597) are shown as boxplots (median, 25th and 75th percentiles (edges of the boxes) and whiskers for extreme values not considered as outliers (o)). Statistically significant differences between the groups are indicated by asterisks (Mann Whitney test, p < 0.001). (**B**) Inter-path differences shown for every 1 m along the tree-to-nest distance. Red, intra-ant differences (n = 56). Blue, inter-ant differences (n = 1,597). Lines show the medians for every metre along the tree-to-nest distance, and lower and upper edge of the shaded areas show the 25th and 75th percentiles, respectively. Mann Whitney tests: tree-to-nest distance; 1 m; p > 0.05 (ns); tree-to-nest distance, 2 m, p < 0.01 (*); all other tree-to-nest distances, p < 0.001 (**)

To test for idiosyncrasy in the homing routes of wood ants navigating along shared odour trails in complex woodland environments, we recorded three consecutive homing paths of 20 individual foragers in their natural habitat (Fig. 1). Figure 2 shows how the paths from individual ants are similar to each other when compared to the spread of paths from all ants. Quantitatively, we show that the mean inter-path differences were significantly smaller within individuals than between individuals (Mann Whitney test, Z = 8.2, *p* < 0.001, Fig. 3A). Hence, the three paths from an individual ant were more similar to each other than to routes from other ants, indicating that indeed individual wood ants do follow idiosyncratic routes (Fig. 2) when travelling from the foraging tree to their nest.

We further looked at the evolving intra- and inter-ant route differences for every metre along the tree-to-nest direction. This showed that intra-ant comparisons were significantly different from inter-ant comparisons for all distances after 1 m along their homing paths (Fig. 3B; Mann Whitney tests by tree-to-nest distance: 1 m, *p* > 0.05; tree-to-nest distance, 2 m, *p* < 0.01; all other tree-to-nest distances, *p* < 0.001). Ants shared an approximate starting point at the base of their foraging tree, and looking at the entire set of foraging paths (Figs. 2 and 3B) revealed that individual ants have chosen unique paths as they spread out from this point. The paths of individual ants (Fig. 3B), however, show a stable inter-path difference along the entire homing distance, suggesting they are applying guidance strategies that control paths to maintain a habitual route.

**Fig. 4:**
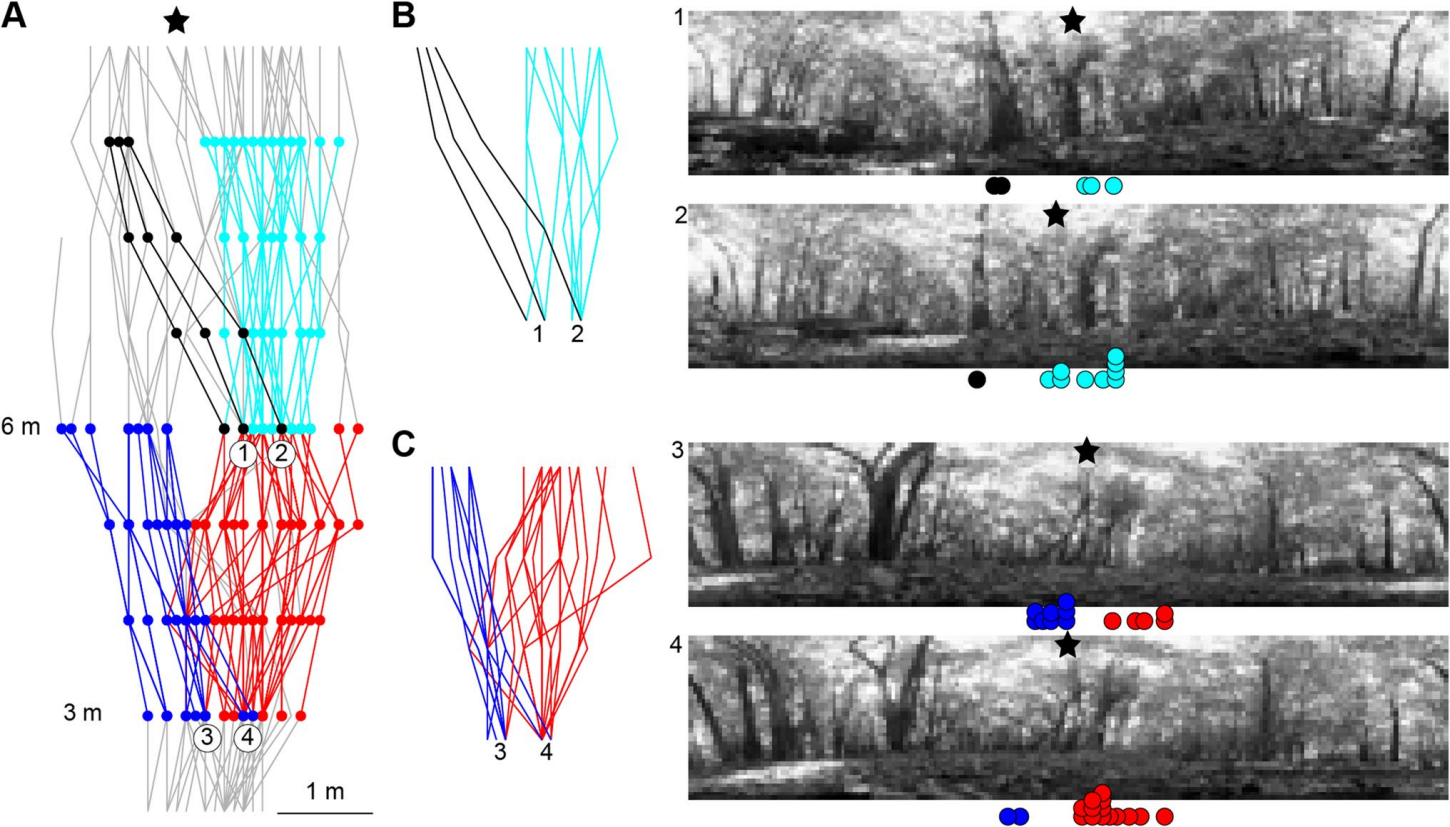
Ant’s eye views from route divergence points. (**A**) All paths from Fig. 2 are shown in grey (trimmed here at 2 m and 10 m). The asterisk indicates the nest direction. At 3 m along the tree-to nest direction, five ants consistently navigate to the left (shown in dark blue), whereas 13 ants consistently navigate to the right (shown in red). From the ants travelling to the right, one ant then navigates consistently to the left (shown in black) and 11 ants navigate consistently to the right (shown in light blue) when they reach 6 m. (**B**) Path sections between 6 m and 9 m from (A) are shown for two selected locations. At 6 m, two ant’s-eye-view pictures are shown for the highlighted locations (1) and (2). The asterisk indicates the nest direction. Coloured circles below the picture show the heading directions from 6 m to 9 m for the coloured paths in their respective colours. (**C**) Path sections between 3 m and 6 m from (A) are shown for two selected locations. At 3 m, two ant’s-eye-view pictures are shown for the highlighted locations (3) and (4). The asterisk indicates the nest direction. Coloured circles below the picture show the heading directions from 3 m to 6 m for the coloured paths in their respective colours

Figure 4 shows the specific directions taken by individual ants relative to the panoramas experienced. At 3 m along the tree-to-nest direction, five ants consistently navigated to the left of the trail, whereas 13 ants consistently navigated to the right of the trail (Fig. 4A). From the ants travelling to the right, one ant then navigated consistently to the left, and 11 ants navigated consistently to the right when they reached 6 m (Fig. 4A). The ant’s eye view panoramas reveal that there are subtle differences in the views for nearby locations at 3 m (Fig. 4C). At 6 m, an object on the ground visible on the left in the picture, probably a small piece of tree trunk on the ground, means that there are big changes in the view for nearby locations on the 6-m line (Fig. 4B). Differences in the panoramas from nearby locations on the 3-m and 6-m lines shows that there is visual information that individual ants can use to direct the different headings taken from those points.

## Discussion

We show here that trail-using wood ants follow idiosyncratic routes in complex landscapes (Figs. 2 and 3). We suggest that individual ants learn specific spatial information to control their routes, despite pheromone trails being available. This learnt information provides redundant spatial information alongside the information the ants have from the pheromone trails and path integration. This raises fascinating questions about the interactions between personal and collective navigational processes, the nature of the specific sensory cues that underpin learnt idiosyncratic routes in complex habitats, and whether there is a memory versus accuracy trade off in navigation.

### Sensory ecology of collective and individual navigation in ants

Wood ants learn and follow idiosyncratic routes when travelling back and forth along trunk trails in the cluttered woodland habitat. Hence, the foragers use individually acquired spatial knowledge to navigate along the collective odour trail. Each ant species experiences a different sensory ecology for navigation (Knaden & Graham, 2016), which influences the available spatial information and the optimal balance of cues (Buehlmann et al., 2020b). Often, a clever combination of innate and learnt navigational strategies allows ants to robustly navigate through the world (Buehlmann et al., 2020b). Innate strategies such as path integration or pheromone trails act as a scaffold that enables the learning of other sensory cues useful for navigation (Buehlmann et al., 2020b). For instance, in the wide and open spaces of the North African desert environments, solitarily foraging *Cataglyphis* species are equipped with prodigious abilities to navigate via path integration (Wehner, 2009). Integrating directional information derived from celestial cues (Wehner & Mueller, 2006) and information about the distance travelled from a step counter (Wittlinger et al., 2006) allows ants to have a constant read-out of their position relative to the starting point of a foraging journey (Mueller & Wehner, 1988). Even though these desert ants rely heavily on path integration, they still learn and use visual or olfactory information when available (Buehlmann et al., 2015; Steck et al., 2011; Wehner et al., 1996). For example, path accuracy is increased when ants combine path integration with spatial information provided by the nest odour (Buehlmann et al., 2012), the distant visual panorama (Huber & Knaden, 2015), or their nest hill that can act as a natural visual landmark (Freire et al., 2023). Other desert ant species inhabit more cluttered low-shrub and grassland terrains and demonstrate rapid learning of the visual information provided by the natural vegetation (Buehlmann et al., 2011; Haalck et al., 2023; Mangan & Webb, 2012; Wystrach et al., 2011b). These desert ants learn and follow visually guided idiosyncratic routes between the nest and a feeding site (Kohler & Wehner, 2005; Mangan & Webb, 2012). Path integration runs constantly and is essential when ants are unfamiliar with an environment, however, visual route guidance is so robust that once developed it can be used even when it is at odds with path integration (Kohler & Wehner, 2005; Mangan & Webb, 2012). In even more cluttered environments, ants that depend on trees for foraging opportunities have to navigate through complex three-dimensional environments where the tree canopy can block the view of celestial compass information (e.g. Macquart et al., 2006; Narendra et al., 2013; Rosengren, 1971), hence making the use of path integration challenging. The sensory ecology of some of these species is different to desert ants because of the use of social information via pheromone trails on shared foraging trails, with our study species being one such ant that uses social odour trails for recruitment to food sources (Rosengren & Fortelius, 1987). Like path integration, pheromone trails allow ants to learn environmental cues while they are safely connected to the nest. In the laboratory, wood ants have been shown to use a range of sensory cues, such as simple visual (Buehlmann et al., 2016; Graham et al., 2003; Lent et al., 2013) and non-pheromone olfactory (Buehlmann et al., 2020a) cues, as well as some aspects of path integration (Fernandes et al., 2015); however, we have little understanding of how they navigate in the densely cluttered woodland habitat. Our new results show that wood ants repeatedly follow the same routes instead of choosing a spread of paths within their shared trail, hence, acquiring individual spatial information for navigation.

### Following idiosyncratic routes using ground based sensory cues

The idiosyncratic routes observed in wood ant foragers demonstrate that individual ants do not choose a trajectory at random on their trail, but that they have learnt specific sensory information to guide individual paths. We observed high levels of foraging traffic with hundreds of wood ants along the approximately 3-m wide trail between the nest and the tree (Fig. 2). Following collective pheromone trails is useful for ants that are inexperienced or unfamiliar with an environment, because such a trail can act as a scaffold for individual route learning (Collett et al., 2003). However, this shared trail cannot guide the ants’ idiosyncratic routes with the precision seen in this study. Similarly, it is unlikely that individual foragers are depositing and following personal pheromone depositions. Hence, spatial information from pheromone trails is not sufficient to guide individual and stereotypical routes as seen in our current study. Sensory cues for guiding the ants’ paths could be associated with the woodland floor, such as gradients, texture, ground structure and non-pheromone odours. Ants are known to learn and use non-social odours to guide routes (Buehlmann et al., 2020a; Buehlmann et al., 2015), and odour analyses of desert ant habitat show that there are useful place-specific concentration gradients of environmental odours (Buehlmann et al., 2015), potentially allowing ants to develop stable olfactory-guided routes. In addition to smells, ground textures have also been shown to be learnt and used as landmarks to accurately pinpoint a goal (Seidl & Wehner, 2006). We have anecdotal observations where ants made small-scale path corrections on the basis of small branches on the ground, suggesting that ground structures do play some role in route guidance in wood ants. However, we suggest that the primary sensory cue guiding idiosyncratic routes are visual, where stored views for guidance are specific to small regions of space, such that specific instructions can be differentiated for different parts of the route (Wystrach et al., 2012). Ants treat large areas as the same place when they are in open areas (i.e., locations appear very similar to ants), whereas cluttered areas contain more different views allowing ants to control for more frequent changes of direction (Wystrach et al., 2019). Over the last couple of decades, we have learnt that wood ants are excellent visual navigators, at least in laboratory scenarios (Buehlmann et al., 2016; Graham et al., 2003; Lent et al., 2013). Similarly, a long-standing strand of research on a variety of ant species has implicated vision for route guidance (Collett et al., 2014; Macquart et al., 2006; Mangan & Webb, 2012; Narendra et al., 2013; Rosengren, 1971) when other cues are available. Finally, it is also worth mentioning that ants have innate biases that can be seen at the population level (wood ants: Buehlmann & Graham, 2022; Graham et al., 2003; Voss, 1967). However, this cannot explain the fact that ants follow individual routes. This could be due to individual biases that are unique to individual ants (Zoltan, 2021), i.e., ants could have individual biases rather than individually learnt routes. Our data alone cannot yet fully answer the question of how wood ants control and guide their idiosyncratic routes. However, idiosyncratic routes of wood ants recorded in the lab have been shown to be controlled by visually learnt sequences (Harris et al., 2007), which suggests that individual visual memory is used in wood ants following idiosyncratic routes.

### Memory versus accuracy trade off in the use of proximal and distal visual cues

For visual route guidance, ants use the information from large portions of visual scenes or even entire panoramas (Graham & Cheng, 2009; Wystrach et al., 2011a; Wystrach et al., 2011b), remembering the egocentric scene to act as a ‘visual compass’ when setting route directions (Baddeley et al., 2012; Collett, 2014; Schwarz et al., 2017; Wystrach et al., 2012; Zeil, 2012). Lots of research investigating visual navigation in ants’ natural habitats has focused on desert ants inhabiting low-shrub and grassland deserts (Christian & Morton, 1992; Muser et al., 2005). Wood ants investigated here, however, inhabit dense woodlands where the tree canopy often fills the view to the sky. This suggests that wood ants might use the canopy pattern for navigation as described for ponerine ants inhabiting the tropical forest (Hoelldobler, 1980). In such cluttered environments, sensory cues, including vision, provide not only a complex but also sometimes an intermittent signal. Uneven terrain means that access to visual scenery might be temporarily interrupted when beneath dense undergrowth. However, ants were previously shown to have robust path guidance by keeping stable heading directions over short periods of time when visual cues are temporarily out of sight (Ardin et al., 2016; Lent et al., 2009; Pfeffer et al., 2016; Schwarz et al., 2017) or the visual panorama is distorted (Schwarz et al., 2014). We suggest that route guidance in wood ants navigating through cluttered terrain comes from a combination of visual direction setting and course control mechanisms.

One other problem faced by visual navigators is to balance the storage and use of visual information from close and far objects. Distal visual cues can function as a visual compass and are useful for maintaining a heading direction over a large distance. This is memory efficient but does not allow ants to accurately follow idiosyncratic routes, which require frequent changes of direction. Proximal visual cues allow a high path accuracy (Schultheiss et al., 2013; Zeil et al., 2003), and are thus more useful in some situations (Wystrach et al., 2019), but it means that a high number of views need to be memorised because proximal cues change rapidly. Hence, there is a trade-off between accuracy and memory load. Recent computational modelling suggests that ants only need a fraction of their memory capacity to learn views around their nest during learning walks (Wystrach, 2023); however, there is little understanding of how costly it is to store views. The specific guidance instructions for individual ants relative to the panoramas shown in Fig. 4 reveal that ants do experience both subtle and large changes in panoramic views over small distances. This can explain how different ants can use unique visual memories to guide routes in different directions from the same location, but also indicates the idiosyncratic routes would incur a high visual memory cost (Buehlmann et al., 2020c; Kamhi et al., 2020).

## Conclusion

Taken together, we show here that wood ants use individually acquired spatial knowledge when following idiosyncratic routes along trunk trails. We suggest that visual memories are guiding those routes; further studies, however, need to investigate in more detail how different sensory modalities are integrated in complex natural landscapes. More generally, we need to expand our knowledge of how behaviour is shaped by the animal’s sensory ecology and ultimately also how brains and sensory systems are adapted to produce such natural behaviour.
